# From Individual Expression to Group Polarization: A Study on Twitter’s Emotional Diffusion Patterns in the German Election

**DOI:** 10.3390/bs15030360

**Published:** 2025-03-13

**Authors:** Yixuan Zhang, Bing Zhou, Yiyan Hu, Kun Zhai

**Affiliations:** 1 School of Foreign Studies, University of International Business and Economics, Beijing 100029, China; 202100720118@uibe.edu.cn; 2 Department of Linguistics, University of Vienna, 1010 Vienna, Austria; 3 Division Digital Philology, Department of European and Comparative Literature and Language Studies, University of Vienna, 1010 Vienna, Austria; 4 School of International Development and Cooperation, University of International Business and Economics, Beijing 100029, China; 03247@uibe.edu.cn (B.Z.); 202412012541@uibe.edu.cn (Y.H.); 5 School of International Studies, Peking University, Beijing 100871, China

**Keywords:** group emotion, derogatory speech, emotional diffusion, social media polarization

## Abstract

This study analyzes 194,151 tweets from the 2021 German federal election using sentiment analysis and statistical techniques to examine social media’s role in shaping group emotions, voters’ emotional expression and derogatory speech toward candidates, and the relationship between sentiment intensity and tweet spread. The findings show that negative emotions dominated social media discussions. Additionally, voter perceptions towards candidates on social media also follow a pattern of negativity, often characterized by derogatory speech. This takes four main forms: intelligence-based attacks, animal metaphors, character insults, and gender-based discrimination, with female candidates disproportionately affected. Moreover, the study finds that negative emotions exhibit significantly greater diffusion and reach compared to positive and neutral sentiments on social media. This study further examines election fairness and political dialog openness through the lens of equity, inclusion, diversity, and access (IDEA). These findings emphasize individual and collective emotional dynamics in the social media environment, highlighting the need for governance strategies that promote equity, inclusivity, and diversity in digital political discussions.

## 1. Introduction

The 2021 German federal election was a major turning point in the country’s political landscape, marking the end of Angela Merkel’s 16-year tenure as Chancellor. The election was highly competitive, with key debates focusing on climate policy, economic recovery after COVID-19, and Germany’s future role in the European Union.

Under Olaf Scholz, the Social Democratic Party (SPD) won 25.7% of the vote, making it the most voted party. Scholz campaigned on stability, economic reform, and social justice, positioning himself as Merkel’s pragmatic successor. Other key contenders included Armin Laschet of the Christian Democratic Union/Christian Social Union (CDU/CSU), Annalena Baerbock of the Green Party (the first female Green Party candidate for Chancellor), Christian Lindner of the Free Democratic Party (FDP), and Alice Weidel of the far-right Alternative for Germany (AfD). In the end, the SPD successfully formed a “traffic light coalition” with the Greens and FDP, becoming the new governing alliance.

As a result of social distancing measures and restrictions on traditional campaigning during the COVID-19 pandemic, it was considered the most digitalized election in history, with social media playing a major role as one of the main platforms (Sältzer & Stier, 2022). During the election, emotional expression on social media was a widely discussed phenomenon. Some studies suggest that social media platforms may contribute to emotional segmentation during elections. This could influence public perceptions and attitudes toward candidates. However, the extent and mechanisms of this influence remain subjects of ongoing academic debate (Stier et al., 2020). Therefore, analyzing Twitter data from the 2021 German federal election helps to understand the characteristics of group emotions, the patterns of voters’ emotional expressions toward candidates, and the dynamics of emotional diffusion.

As social media becomes a core channel for election communication, it has a dual impact on group interactions and individual voter perceptions. On one hand, social media provides unprecedented interactivity, allowing candidates to engage directly with the public and making political discussions more immediate and accessible (Stier et al., 2018). On the other hand, social media functions not only as an information-sharing platform but also as a medium for emotional expression. Some studies suggest that social media algorithms may prioritize emotionally charged content over objective information, potentially influencing user exposure to information (Flaxman et al., 2016). Additionally, the widespread circulation of misinformation (Vosoughi et al., 2018) and the potential manipulative effects of micro-targeted political advertising (Bodó et al., 2017) were suggested to influence voter perceptions. Some scholars argue that emotional bias on social media might challenge the rational foundation of democratic discourse (Duncombe, 2019). Despite the European Union’s regulatory efforts (Gorwa, 2019), digital democracy remains vulnerable, and the fairness of the election information environment faces ongoing challenges.

Although the role of social media in political communication has gained increasing attention, existing studies primarily focus on candidate image building and public engagement on social media (Klinger & Svensson, 2015; Tucker et al., 2018). However, research on the characteristics and diffusion of sentiment during elections remains limited (Bossetta, 2018), particularly regarding group polarization and the patterns of derogatory speech and their spread, which are important yet still underexplored topics.

Based on 194,151 tweets from the 2021 German election, this paper combines sentiment analysis techniques to answer the following questions:

**Q1:**  *How do individual emotions evolve into group emotions in social media discussions during the election?*
**Q2:**  *How do voters express emotional perceptions of candidates on social media? What patterns and characteristics emerge in derogatory speech within election discussions?*
**Q3:**  *Does sentiment intensity correlate with the spread of tweets? Are negative tweets more widely disseminated than positive or neutral ones?*

**Figure 1:**
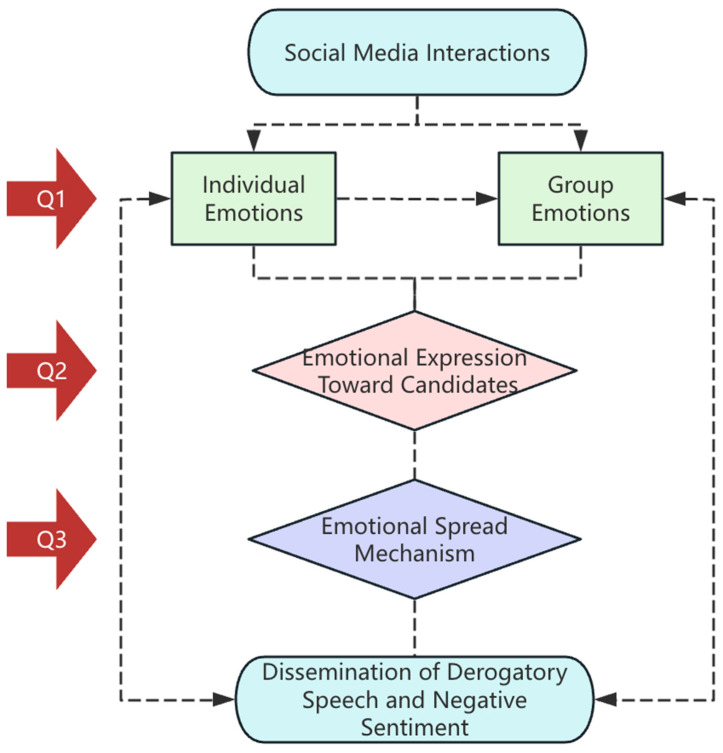
Research model.

This model (Figure 1) is designed to address the three core research questions of this study, illustrating its overall structure. As interactions within social networks increase, individual emotions have the potential to merge into group emotions. On this basis, voters’ emotional expressions toward candidates exhibit certain patterns, typically characterized by derogatory speech. Additionally, this study explores the impact of sentiment tendencies and intensity on the dissemination of tweets. This research may provide comparative insights for 2025 and future elections, enabling cross-national comparisons with other electoral contexts (Ferrara & Yang, 2015).

## 2. Literature Review

### 2.1. Research on the Application of Twitter in Elections

Candidates, journalists, and the public increasingly use Twitter during election campaigns, leading to increasing research on its role in political elections. Since this study focuses on Twitter’s during the 2021 German federal election, research on Twitter’s role in other political contexts—such as in civil protests, wartime communication, and government outreach—was excluded. In the literature review, studies are categorized into two main areas: the candidates’ use of Twitter and the public’s use of Twitter (Table 1).

#### 2.1.1. Research on Political Parties’ and Candidates’ Use of Twitter

Research on this topic focuses on three key questions: Which political parties and candidates are more likely to use Twitter? What functions of Twitter do they primarily use? What impact does Twitter have on their campaigns?

Regarding the first question, opposition parties and their candidates are more inclined to use Twitter than members of the ruling party (Hemphill et al., 2013; Lassen & Brown, 2011; Peterson, 2012; Plotkowiak & Stanoevska-Slabeva, 2013; Shogan, 2010; Vergeer & Hermans, 2013). In terms of age, younger politicians are more likely to use Twitter than older politicians (Lassen & Brown, 2011; Peterson, 2012; Vergeer & Hermans, 2013; Jackson & Lilleker, 2020; Straus et al., 2013). Regarding the impact of gender on Twitter usage, research findings remain inconclusive. Some studies suggest that male candidates are more likely to use Twitter (Hemphill et al., 2013), while others argue that female candidates are more active users (Jackson & Lilleker, 2020; Evans et al., 2014; Larsson & Kalsnes, 2014). Additionally, the frequency of Twitter usage often correlates with the intensity of electoral competition (Evans et al., 2014). When members of a political party have previously succeeded in using Twitter, new candidates from the same party are more likely to adopt it (Chi & Yang, 2011).

Second, regarding how political parties and candidates use Twitter, most studies use content analysis, interviews, or quantitative methods to examine tweets posted by parties and candidates. Many studies analyze certain Twitter functions, such as mentions (@), retweets, and hyperlinks. Research indicates that parties and candidates primarily use Twitter to publish campaign-related information and link to their official websites (Graham et al., 2013; Graham et al., 2016; Macnamara, 2011; Small, 2010; Evans et al., 2014). Candidates’ explicit calls for action, such as mobilizing voters, are relatively rare (Hemphill et al., 2013; Evans et al., 2014; Golbeck et al., 2010).

In terms of communication style, ruling party candidates tend to use Twitter in a broadcasting manner, with limited interaction with other users (Shogan, 2010; Jackson & Lilleker, 2020; Evans et al., 2014; Graham et al., 2013; Graham et al., 2016; Macnamara, 2011; Small, 2010; Adams & McCorkindale, 2013; Baxter & Marcella, 2012; Shapiro & Hemphill, 2017; Grant et al., 2010). Opposition candidates, on the other hand, are more likely to interact with voters (Jackson & Lilleker, 2020; Larsson & Skogerbø, 2018). Moreover, interactions between politicians predominantly take place within the same party rather than across party lines (Livne et al., 2011; Jürgens & Jungherr, 2015).

Regarding the impact of Twitter usage on candidates, research findings vary. First, compared to television and newspaper appearances, posting tweets can increase candidates’ perceived connectedness and social presence, particularly when they share personal and informal content. This suggests that candidates and parties intentionally use Twitter to present themselves as modern and relatable figures (Kreiss, 2016).

Furthermore, studies on U.S. elections suggest that candidates use Twitter to apply for small-scale political donations, thereby increasing overall campaign funds (Allcott et al., 2019; Song, 2014). However, regarding whether Twitter usage contributes to electoral success, research indicates no clear correlation between the two (Vergeer & Hermans, 2013).

#### 2.1.2. Research on Public Use of Twitter in Elections

Research indicates that only a small portion of the public actively participates in election discussions on Twitter. Among these politically engaged users, younger individuals, male users, and students tend to be more active (Vaccari et al., 2013). Furthermore, opposition party supporters are more likely than ruling party supporters to express their opinions on Twitter and post more frequently (Straus et al., 2013; Conover et al., 2012).

In terms of information dissemination, while social media has ruined the traditional one-way flow of information and has given every user equal rights as content producers, significant differences remain concerning influence and reach between ordinary users and opinion leaders. The core figures in Twitter’s interaction network play an important role in filtering and distributing information. In addition, traditional media accounts still dominate on Twitter, generating high engagement in terms of retweets and comments, which reflects the strong connection between traditional and digital media (Morstatter et al., 2014).

Overall, the number of tweets tends to increase over time, peaking near the end of the election campaign. Significant political events related to candidates or campaigns also lead to short-term spikes in Twitter activity (Rimé et al., 1991). Regarding content, discussions focus more on political leaders and their electoral behavior rather than on political issues or election platforms (Pang & Lee, 2008).

### 2.2. Application of Sentiment Analysis in Social Media Research

Social media has become a key platform for emotional expression, diffusion, and resonance. Therefore, the application of sentiment analysis in social media research is crucial for understanding the individual emotions, group emotions, and their spreading mechanisms.

Sentiment analysis, also known as opinion mining, is a widely used technique for identifying and evaluating emotions in textual data. It classifies sentiments into positive, negative, or neutral categories, helping to understand public opinions on specific topics (Yu et al., 2013; Oliveira et al., 2017). Sentiment analysis extracts subjective information from large amounts of unstructured data by integrating data mining, machine learning, natural language processing, and information retrieval (Oliveira et al., 2017). Researchers have applied this approach to fields including politics, finance, and healthcare to assess trends and public sentiment (Banda et al., 2021; Kontopoulos et al., 2013).

Sentiment analysis is conducted at three main levels: the document-level, sentence-level, and aspect-level. Document-level analysis determines the overall sentiment of an entire text, sentence-level focuses on individual sentences, and aspect-level identifies emotions related to specific topics within the text (Thomas et al., 2006; Wong et al., 2008). Machine learning-based sentiment analysis has proven to be highly effective, using algorithms to classify large datasets by learning patterns from labeled data (Ghiassi et al., 2013).

The applications of sentiment analysis play a crucial role in political discourse analysis and crisis management (Wong et al., 2008; Lamy et al., 2016). In political studies, researchers have compared sentiment analysis results with traditional opinion polls to assess its predictive power in elections (Oliveira et al., 2017). Additionally, Twitter-based sentiment analysis has been widely used to understand public reactions to political events, such as elections and policy changes (Ghiassi et al., 2013). Researchers use Twitter data to assess public sentiment toward candidates and policies, aiming to predict election outcomes. Jahanbakhsh and Moon (2014) analyzed 32 million tweets from the 2012 U.S. presidential election and found that sentiment analysis could evaluate candidates’ popularity and compare it with traditional polling results. Additionally, Karami et al. (2018) introduced a computational approach combining sentiment analysis and topic modeling to examine public discussions on economic issues during the 2012 U.S. presidential election. Their findings suggest that Twitter data can help reveal public sentiment and key concerns on specific topics. However, not all studies have proved a direct connection between Twitter sentiment and election results. Singh et al. (2022), in their study on the 2020 Delhi Assembly elections, observed that while candidates’ Twitter activity correlated with election outcomes, the frequency of party mentions and sentiment trends did not directly align with the final results. This indicates that although Twitter sentiment analysis provides valuable insights into voter emotions and election predictions, the findings remain inconsistent. Further research is needed to evaluate the effectiveness and limitations of using Twitter data for sentiment analysis in electoral studies.

### 2.3. Ideological Homophily, Emotional Diffusion, and Echo Chambers in Social Media

The formation of ideologically homogeneous online networks, commonly referred to as echo chambers, is considered a key mechanism contributing to online political polarization.

Individuals’ tendency toward ideological homophily, combined with algorithmic amplification, may reinforce the dominance of emotional and negative discourse in election discussions. Prior (2013) found that users tend to select information that aligns with their existing views in both traditional and social media environments, and social media’s interactive features may further amplify this trend. Boutyline and Willer (2017) and Del Vicario et al. (2017) showed that social media networks primarily consist of users with similar political views. This ideological homophily may limit exposure to diverse perspectives, leading users to engage mostly with content that reinforces their prior beliefs. Additionally, social media platforms’ algorithmic recommendation systems tend to prioritize content that triggers strong emotional reactions (Barberá, 2020), particularly content associated with anger or hostility (Del Vicario et al., 2016). This suggests that social media does not distribute information randomly; instead, it favors emotionally charged content through the combined effects of user preferences and platform algorithms.

Like-minded group interactions may reinforce political views and contribute to emotional contagion and conflict escalation during elections. Sunstein (2002) described group polarization, where individuals who engage primarily with like-minded peers tend to adopt more extreme positions. Bright (2018) further noted that extremist content is often more provocative, making it more likely to attract attention and gain traction on social media. This suggests that social media is not only a space for political discussion but also a platform that may encourage emotional mobilization and political confrontation. This dynamic becomes especially visible during elections. Yurtcicek Ozaydin (2021) found that hashtags often serve as tools for political confrontation, with different political groups using the same hashtags to push opposing messages, creating “hashtag wars”. This intensifies partisan divisions and may influence how voters perceive candidates, contributing to increased political polarization.

In summary, political divide on social media during elections may result from the combined effects of ideological homophily, algorithmic content amplification, and the characteristics of emotional diffusion. Users tend to engage with content that aligns with their views, while platform algorithms may amplify emotionally charged content, making negative discourse more prominent in election discussions. At the same time, like-minded group interactions may strengthen political views, making emotional discourse more provocative and, in some cases, escalating political divisions. These mechanisms together shape the social media environment during elections, making political discussions more confrontational and potentially deepening political polarization.

## 3. Research Methods

### 3.1. Mixed Methods

Mixed methods research integrates quantitative and qualitative approaches, leveraging the strengths of both methodologies. Creswell and Clark (2017) noted that mixed methods combine the objectivity and broad scope of quantitative research with the depth and contextual understanding of qualitative research. Tashakkori and Teddlie (1998) further highlighted that this approach enhances the explanatory power of research findings and effectively addresses complex research problems. In this study, mixed methods are particularly suitable for analyzing large-scale data while allowing for a nuanced exploration of specific negative emotions. The combination of macro-level statistical analysis with micro-level interaction studies enables a more comprehensive understanding of emotional expressions. Quantitative methods help identify overall trends in Twitter discussions, while qualitative methods provide deeper insights into social interactions and emotional expressions within specific contexts (Bryman, 2006).

### 3.2. Data Collection

This study focuses on all German-language tweets related to the 2021 German federal election posted on Twitter. Given that the voting date for this election is 26 September 2021, this study chooses the month of the election as the time range, collecting Twitter data from 30 August 2021, to 30 September 2021.

**Figure 2:**
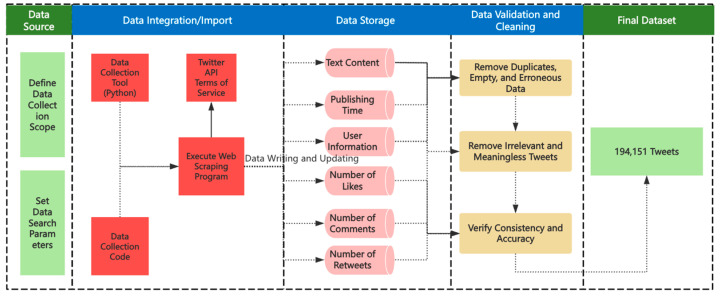
Data collection process.

This study follows a structured data collection process to ensure the scientific validity and reliability of the dataset (Figure 2).

1. **Defining the Data Collection Scope and Search Parameters:** The data collection time range is from 30 August to 30 September 2021. The dataset includes German-language tweets related to the election, capturing tweet content, usernames, timestamps, number of comments, retweets, likes, and mentions (@) and hashtags (#) used in the tweets. Before data collection, keywords and search parameters were set to ensure topic relevance. The selected keywords include “*Bundestagswahl 2021”, “*btw21”, and their common variants to cover election-related discussions.
2. **Accessing Twitter Data:** Since the Twitter API has certain limitations in terms of time range, this study uses a third-party web scraping tool (Python 3.10.10) to collect data.
3. **Executing the Web Scraping Program:** Based on the predefined search parameters, a web scraping program was used to collect tweets. The dataset includes tweets from 55,429 users, with a total of 272,548 tweets collected.
4. **Data Verification and Cleaning:** After data collection, a comprehensive cleaning process was conducted. First, duplicate tweets, null data, and obviously erroneous data were removed; second, tweets with meaningless or irrelevant content—such as those containing only images or emojis—were filtered out; finally, the consistency and accuracy of the data were verified. After cleaning, the final dataset consisted of 194,151 valid tweets from 47,090 users.

### 3.3. Sentiment Analysis

This study applies Python and its text analysis libraries, NLTK, to assign sentiment scores to tweets. In order to ensure the accuracy of sentiment analysis, this paper uses the German sentiment dictionary SentiWS (Sentiment Wortschatz). SentiWS is a German sentiment lexicon developed by the natural language processing research team of the University of Leipzig, designed to support sentiment analysis and opinion mining of German texts (Remus et al., 2010). The lexicon contains a large number of words with sentiment scores, which are labeled with their sentiment polarity (positive, negative, or neutral) and scores (expressed as weights of [−1, 1]). It covers four main word classes, *adjectives*, *nouns*, *verbs*, *and adverbs*, and includes their word form changes to adapt to the complex morphological characteristics of the German language. In this study, we use version 1.8b, which includes the following elements (Table 2).

**Figure 3:**
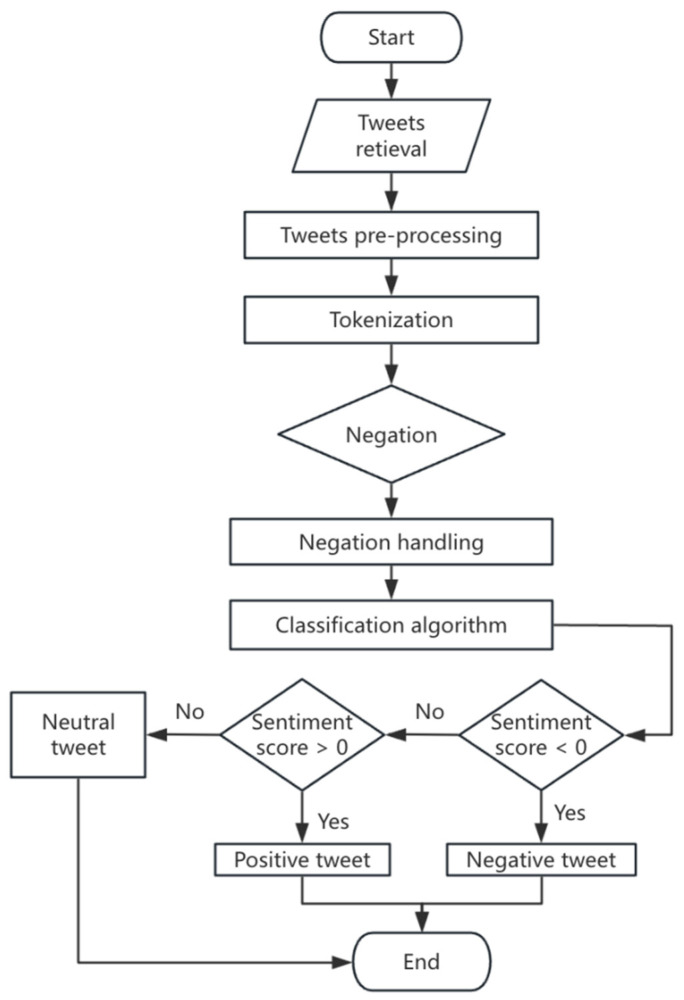
Flow chart for sentiment analysis.

The sentiment scoring process consists of the following steps (Figure 3):

1. **Data Pre-processing:** Tweets are cleaned using regular expressions to remove hyperlinks, emojis, special characters, and irrelevant punctuation. The tweets are then split into words using a word segmentation tool to ensure the correct processing of German grammar.
2. **Word Matching and Sentiment Score Calculation:** Each word in a tweet is matched against entries in the SentiWS sentiment lexicon. If a sentiment word is matched, extract its corresponding sentiment weight and accumulate the sentiment weights of all matching words in the tweet. If the tweet contains negative words (such as “nicht” and “kein”), reverse the sentiment weight within its influence range (Taboada et al., 2011).
3. **Sentiment Score Normalization:** In order to ensure the comparability of sentiment values, the sentiment values of tweets are normalized to the interval [−1, 1], where −1 represents extreme negative sentiment, 1 represents extreme positive sentiment, and 0 represents neutral sentiment. This method avoids the imbalance problem of sentiment values between short and long texts by considering the length of the tweets.
4. **Sentiment scoring criteria:** The sentiment value range of tweets is set to [−1, 1], which is specifically defined as follows (Table 3).

### 3.4. Sentiment and Spread Analysis

This section uses quantitative analysis to examine the relationship between different sentiment types (positive, neutral, and negative) and their spread ability. It further evaluates the impact of sentiment intensity on information dissemination. This analysis applies quantitative methods using GraphPad Prism 10 as the statistical tool. It integrates data management, visualization, and advanced statistical analysis, supporting non-parametric tests, regression analysis, and multi-group comparisons.

#### 3.4.1. Data Sampling

The dataset of this study comes from the results of the sentiment analysis of 194,151 million tweets. To ensure the representativeness of the data and the effectiveness of the analysis, we extracted statistically significant samples from them. The sample size was determined using the standard statistical sample size formula, with a 95% confidence level and a 5% margin of error, resulting in a sample size of 384 tweets.

To further improve the stability of the results and the effectiveness of the group analysis, the sample size for each sentiment category was expanded to 400 tweets, totaling 1200 tweets. The sample was divided into three sentiment groups (Table 4).

#### 3.4.2. The Indicators for Measuring the Spread of the Tweet

In this study, the spread of the tweet is defined as the visibility and popularity of the tweet. In order to more comprehensively and accurately reflect the spread of the tweet, the spread of the tweet is calculated as the average of the number of reposts, likes, and comments. The number of reposts directly reflects the number of times a tweet is further spread, which is an important indicator of the spread of a tweet; the number of likes represents the user’s recognition and support for the content, which indirectly indicates the popularity of the content; and the number of comments indicates the user’s interactive participation and reflects the attractiveness of the tweet in the discussion.

#### 3.4.3. Statistical Method

To explore the impact of emotion on the spread of tweets, this study conducts analyses from the following three aspects: First, the mean spread of the three groups of positive, neutral, and negative tweets is compared. A Kruskal–Wallis test is applied to evaluate whether there are significant differences in dissemination among the three groups. Secondly, the correlation between the sentiment intensity and the spread is analyzed using Spearman’s rank correlation test. This step assesses the extent to which sentiment intensity influences spread. Finally, a linear regression analysis was used to compare the mean spread of tweets with negative and positive emotions to test whether negative emotions were more likely to spread than positive emotions.

## 4. Results

### 4.1. Overall Distribution of Public Sentiment

**Figure 4:**
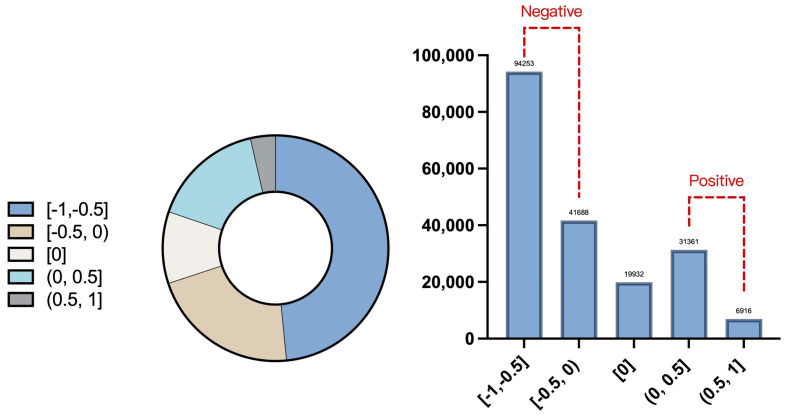
Distribution of sentiment scores.

The descriptive statistics (Table 5 and Table 6, Figure 4) show the sentiment distribution characteristics on social media platforms during the 2021 German election.

From the perspective of the overall sentiment distribution, the average value is −0.30, indicating that discussions on social media were mainly negative. The median of the sentiment score is −0.4, and the 25% quantile is −0.6, further indicating that negative sentiment occupies the main position. At the same time, the standard deviation of 0.4142 suggests there is a certain volatility in the sentiment distribution, but it is not extreme. Most sentiment scores are concentrated near the negative area.

The sentiment interval analysis further verifies the overall trend. In the sentiment interval division, strongly negative sentiment ([−1, −0.5)) accounts for 48.5%, while mildly negative sentiment ([−0.5, 0)) accounts for 21.5%. The total of the two exceeds 70%. The neutral part of the sentiment accounts for only 10.27%, indicating that there are few pure information disseminations or neutral opinions. Mildly positive emotions ((0, 0.5]) account for 16.15%, and strongly positive emotions ((0.5, 1]) account for only 3.56%. Although positive emotions exist, they are far less than the proportion of negative emotions.

**Figure 5:**
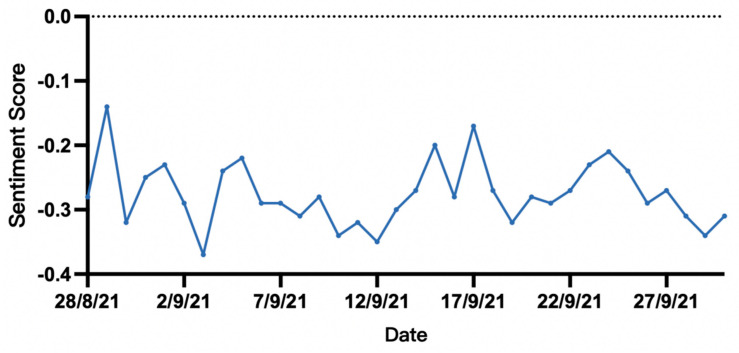
Daily average change in sentiment scores.

From a temporal perspective (Figure 5), public sentiment remained negative throughout the 2021 German federal election period. The mean sentiment score was always below 0, fluctuating between −0.37 and −0.14. Although there were certain ups and downs, the overall trend did not deviate from the negative range.

In conclusion, negative emotions were predominant on social media during the 2021 German federal election, significantly outweighing neutral and positive emotions. Throughout the data collection period, daily sentiment remained predominantly negative. Collective emotions on social media exhibited a strong inclination toward negativity in election discussions. This trend suggests that social media discussions during the election were largely shaped by expressions of dissatisfaction, criticism, and frustration, while constructive or positive discourse appeared less frequently.

### 4.2. Emotional Expressions Toward Candidates and Derogatory Speech

#### 4.2.1. Negative Sentiment Toward Candidates

**Figure 6:**
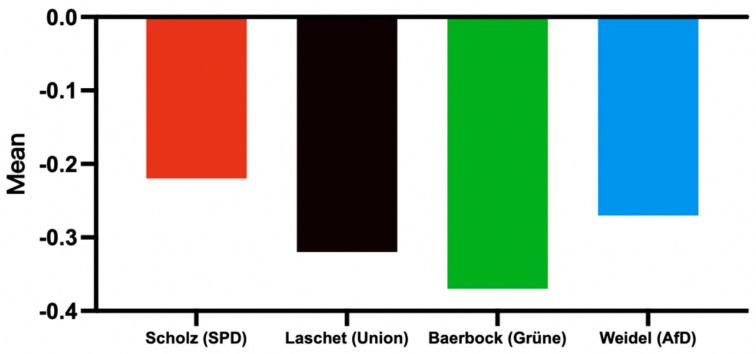
Distribution of candidates’ mean sentiment.

This study found that public expressions of negative sentiment were primarily directed at candidates. This research selects Scholz, Laschet, Baerbock, and Weidel as the core subjects for analyzing negative sentiment. Tweets were categorized based on the candidates they mentioned, and public sentiment scores for each candidate were calculated (Figure 6, Table 7).

In terms of sentiment averages, Baerbock had the lowest sentiment score at −0.372, which is significantly more negative than the other candidates, indicating that she faced the most negative sentiment from the public. Laschet followed closely behind with a sentiment score of −0.325. In contrast, Scholz’s sentiment mean is −0.225, the highest among all candidates, showing a relatively mild emotional image, while Weidel’s is in the middle with −0.275. Overall, Baerbock and Laschet experienced more negative sentiment, whereas Scholz and Weidel maintained relatively milder sentiment profiles.

In terms of the proportion of negative sentiment, Baerbock’s proportion reached 46.13%, which is much higher than other candidates. In terms of positive sentiment, Scholz’s proportion is 8.38%, suggesting a higher level of public support and approval.

#### 4.2.2. Derogatory Speech in Political Discourse

Derogatory language refers to expressions that demean, insult, or belittle individuals or groups, often based on characteristics such as race, gender, sexual orientation, or religion (Cervone et al., 2021). It is particularly prevalent in digital political communication, where social media platforms amplify its reach through algorithmic mechanisms, reinforcing its influence (Barberá, 2020).

In online political discussions, derogatory speech is often used to attack candidates’ competence, credibility, or moral character instead of engaging in substantive debate (Bilewicz & Soral, 2020). A key consequence of derogatory speech is its role in shaping intergroup relations and influencing political polarization. Research suggests that derogatory speech strengthens ingroup favoritism and outgroup hostility, reinforcing pre-existing biases and ideological divisions (Del Vicario et al., 2017; Bilewicz & Soral, 2020). The strategic use of derogatory language in political messaging serves to mobilize supporters, attack opponents, and redefine social norms regarding acceptable discourse (Rossini et al., 2021). This effect is particularly evident online, where algorithmic mechanisms favor emotional content, further increasing the visibility and spread of derogatory speech (Barberá, 2020).

According to the form of attack on candidates, derogatory speech was divided into the following four categories.

The first is intellectual attacks, which attempts to undermine the image of political leadership by devaluing the candidate’s ability and judgment. For example, tweets such as *“Wie kann jemand wie Baerbock so naiv sein? Keine Ahnung von Politik!” (“How can someone like Baerbock be so naive? No clue about politics!”)* and *“Laschet ist einfach inkompetent, eine Katastrophe für Deutschland”. (“Laschet is simply incompetent, a disaster for Germany”.)* reflect the public’s distrust of the candidate’s intelligence level.

The second type is animal metaphor, which incites negative public sentiment by comparing the candidate to certain animals (such as “Schwein” (pig), “Affe” (monkey)), implying behavioral or character flaws. The following are examples of animal metaphors: *“Laschet benimmt sich wie eine Schlange, hinterlistig und gefährlich”. (“Laschet acts like a snake—sly and dangerous”.)* and *“Weidel ist wie ein Schwein im Anzug, keine Klasse!” (“Weidel is like a pig in a suit, no class!”)*.

The third type is attacks on self-esteem. This type of expression attacks the candidate’s moral image by doubting the candidate’s personality or behavior, by using words such as “schamlos” (shameless) and “erbärmlich” (pitiable). Tweets such as *“Laschet ist so erbärmlich, er kann nicht mal eine klare Meinung vertreten”. (“Laschet is so pathetic, he can’t even take a clear stance”.)* and *“Baerbock zeigt sich wieder einmal würdelos, wie erwartet”. (“Baerbock once again shows herself to be undignified, as expected”.)* directly attack candidates’ dignity, reinforcing public dissatisfaction.

The fourth category is gender-based discrimination, which is mainly aimed at female candidates and insults them based on gender role stereotypes. Common terms include “emotional” (emotional), “hysterisch” (hysterical), and “schwach” (weak). Examples such as *“Baerbock ist einfach viel zu emotional, wie kann sie führen?” (“Baerbock is simply too emotional, how can she lead?”)* and *“Frauen wie Weidel gehören nicht in die Politik, sie sind zu schwach”. (“Women like Weidel don’t belong in politics, they are too weak”.)* show stereotypes of emotional instability and weakness to undermine female candidates’ professionalism and leadership image.

The following Table 8 summarizes the main negative sentiment words used against candidates.

This study examines four major candidates—Olaf Scholz, Armin Laschet, Annalena Baerbock, and Alice Weidel—to analyze emotional expression patterns on social media. The findings reveal that female candidates, Baerbock and Weidel, received the highest proportion of negative emotional attacks, with gender-discriminatory remarks being particularly prominent, such as “Baerbock is too emotional to be a leader” or “Weidel is unfit for politics due to a lack of rationality”.

Negative emotions on social media are primarily directed at candidates, with derogatory speech being the dominant form of expression in these discussions. Such language contributes to a more hostile online environment, where criticism often takes the form of insults, sarcasm, or dismissive rhetoric rather than substantive debate. The prevalence of derogatory speech may further degrade the quality of political discourse on social media, potentially intensifying emotional polarization and reinforcing political divisions.

### 4.3. Spread of Tweets and Sentiment Tendency

#### 4.3.1. Significance Testing

To examine differences in the spread of tweets with different sentiment types (positive, neutral, negative), this study applied the Kruskal–Wallis test. Since the data did not meet the assumption of normal distribution and the samples across groups were independent, the Kruskal–Wallis test, a non-parametric method, was more appropriate for comparing the median spread of tweets across the three sentiment groups.

**Figure 7:**
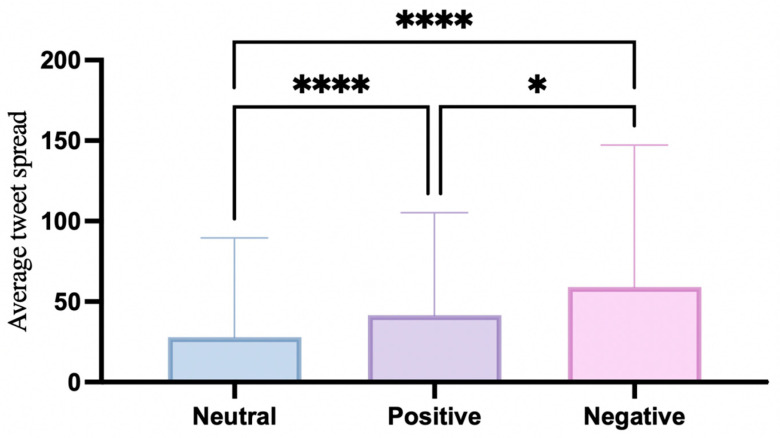
Significance test results. * *p* < 0.05; **** *p* < 0.0001.

The test results (Table 9, Figure 7) show a Kruskal–Wallis statistic of 147.8 with a *p* value < 0.0001. This shows that there are statistically significant differences in the spread of tweets of different sentiment types.

Dunn’s multiple comparison test (Table 10 and Table 11) is further used to clarify which groups show significant differences. Dunn’s test is selected because it corrects for multiple comparisons, preventing potential significance bias. The analysis results show that the spread of different sentiment types is ranked as follows: negative > positive > neutral. Among them, the spread of negative and positive emotional tweets is significantly higher than that of neutral tweets. Although the difference between the spread of positive and negative is small (Z value = 2.578), negative tweets still have a slight advantage.

Overall, emotional tweets and non-emotional tweets show significant differences in their spread. Negative tweets have a clear advantage in dissemination. This trend suggests that negative emotions, such as anger and outrage, are more likely to capture user attention and encourage interaction. Social media interactions further contribute to the spread of negative emotions, aligning with the sentiment distribution pattern observed in Question 1, where negative emotions are predominant.

#### 4.3.2. Correlation Analysis

In order to explore the relationship between the sentiment scores and the spread of positive and negative sentiment tweets, Spearman rank correlation analysis was performed on the two sets of data. The results are as follows (Table 12).

In the positive tweet group, a significant positive correlation was found between sentiment scores and spread (*p* < 0.05). The correlation coefficient is *r* = 0.2523. As sentiment scores increased (i.e., tweets became more positive), the spread also showed a moderate increase. In the negative tweet group, a significant negative correlation was observed between the sentiment scores and spread (*p* < 0.05). The correlation coefficient is *r* = −0.2581, indicating that as the sentiment score decreases (i.e., the more negative the sentiment), the spread increases significantly.

#### 4.3.3. Linear Regression Analysis

**Figure 8:**
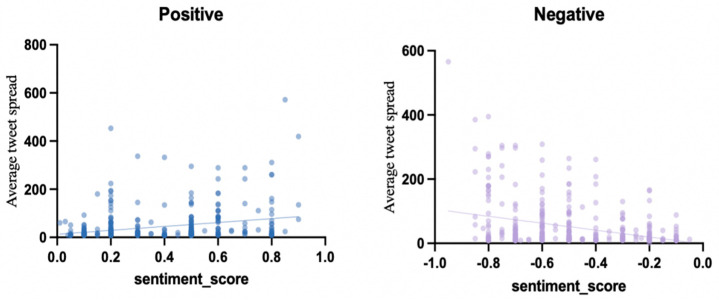
Linear regression analysis.

The results of linear regression analysis (Table 13, Figure 8) show that for the positive tweet group, the regression equation is Y = 80.96X + 12.99, and the slope is 80.96. The positive effect of this sentiment score on the spread is statistically significant. However, the coefficient of determination R^2^ is 0.06986, and only 6.99% of the spread variation can be explained by the sentiment score, indicating that there are many other factors that influence the spread of tweets.

For the negative tweet group, the regression equation is Y = −107.9X − 1.939 with a slope of 107.9, and the coefficient of determination R^2^ is 0.1, which is slightly higher than the positive group, indicating that the negative sentiment score has a stronger explanatory power for the spread, and can explain 10% of the spread variation. This result reflects the slight advantage of negative emotions in promoting spread.

The results show that there are significant differences in the spread of tweets based on the type of sentiment. The negative group has the highest spread rate, significantly exceeding positive and neutral tweets. Spearman rank correlation analysis shows that there is a significant positive correlation between sentiment intensity and tweet spread. Regardless of the sentiment type, the higher the absolute sentiment score (the stronger the emotion), the higher the tweet spread rate. This suggests that emotional content is more likely to attract users’ attention and engagement, thereby promoting spread. These findings also support the “emotion-driven effect” hypothesis.

## 5. Conclusions

This study analyzes 194,151 tweets from the 2021 German federal election, exploring how social media may contribute to the formation of group emotions, the patterns of voters’ emotional expression and derogatory speech toward candidates, and the advantage of negative tweets in terms of spread. In today’s digital political environment, social media is no longer just a platform for information dissemination but a highly emotional interactive network that significantly impacts democratic politics.

First, the study finds that negative sentiment dominated social media discussions, with over 70% of tweets expressing negativity. Specifically, strongly negative tweets (−1 to −0.5) accounted for 48.5%, while mildly negative tweets (−0.5 to 0) made up 21.5%. In contrast, positive tweets (both mild and strong) accounted for only 19.8%, which is significantly lower than the proportion of negative emotions. This reflects the dominant role of emotional discussions on social media platforms, particularly the prevalence of negative sentiment over positive discourse.

Second, the study further explores voter perceptions of candidates on social media and finds that these perceptions also tend to be more emotional, particularly negative. This indicates that voter perception on social media has a tendency toward emotion-based judgments rather than purely rational evaluations. The study further examines the types of derogatory speech, identifying four main forms: intelligence-based attacks, animal metaphors, personality insults, and gender-based discrimination. While the research does not establish a direct causal relationship between emotion and perceptions, it is supposed that prolonged exposure to negative emotional content may contribute to an increasingly extreme and emotionally driven engagement with political topics. Over time, this could create a feedback loop where individuals not only consume but also actively participate in emotionally charged discussions. This process may reinforce polarization and escalating hostility in the digital political sphere.

Additionally, derogatory speech and negative emotions spread far more effectively than rational discussions. The study finds that the intensity of emotions significantly affects the spread of tweets, with strongly emotional tweets reaching a wider audience. Negative tweets are more likely to be shared, triggering group emotional resonance and reinforcing ingroup favoritism. Moreover, due to the confirmation bias effect, voters tend to share information that aligns with their political views, further reinforcing their group identity. This can intensify negative perceptions of opposing groups, contributing to the development of echo chambers and exacerbating political polarization.

Overall, this study explores the sentiment diffusion mechanisms of social media during elections and examines its potential role in emotional polarization and derogatory speech. In the digital democracy environment, the spread of negative emotions and derogatory speech may pose challenges to the quality of democratic discussions during elections. Future research and policy efforts should focus on balancing freedom of expression and emotional regulation on social media to foster a more inclusive, rational, and sustainable democratic dialog.

## 6. Discussion

The study finds that emotions on social media are not expressed in isolation; rather, emotional content spreads rapidly through interactive mechanisms, reaching a wider audience. During election discussions, negative emotions dominate, taking precedence over policy-oriented rational discourse. Perceptions towards candidates also follow a pattern of negativity, often characterized by derogatory speech.

From the IDEA perspective, social media’s communication mechanisms impact the equity of the electoral process. The amplification of negative emotions affects certain candidates, especially women, who are more likely to become targets of attacks and derogatory speech. The study finds that Baerbock and Weidel received a high proportion of gender-discriminatory attacks, such as being described as “too emotional to be a leader”. This suggests that social media is not just an information platform but also shapes the competitive environment of elections, putting some groups at a disadvantage in public discourse. This emotion-driven bias intensifies information inequality, potentially influencing election outcomes by making it harder for certain candidates to gain public trust.

Furthermore, social media’s communication patterns challenge the inclusivity and diversity of political discourse. A public sphere dominated by negative emotions makes it harder for moderate and rational perspectives to gain attention, pushing political discussions toward confrontation. Voters with similar views tend to cluster within homogeneous information networks, creating echo chambers that weaken dialog between opposing perspectives and limit the expression of diverse political voices. Social media algorithms may prioritize certain viewpoints while limiting the visibility of opposing perspectives, potentially constraining open democratic dialog and reinforcing social divisions.

Access to information is also a key factor influencing electoral fairness. Emotion-driven dissemination can make it more challenging for voters to access comprehensive and diverse information, increasing their exposure to emotionally extreme content. Groups with limited access to information often rely on social media as their primary source of political news, which may make them more susceptible to developing one-sided views within echo chambers. This dynamic can further impact the quality of democratic discourse, shifting political narratives away from factual discussions and toward emotional bias and polarization.

This study still has certain limitations. First, the data used in this study are only derived from Twitter, which does not represent all voters. Future research could incorporate data from other social platforms to provide a more comprehensive understanding of sentiment transmission. Second, the sentiment analysis method relies on a dictionary-based approach. While effective for large-scale data processing, it may have limitations in accurately identifying emotional tendencies in irony, humor, or complex contexts (Zhang et al., 2018). Future studies could integrate deep learning techniques to enhance analytical accuracy. Additionally, this study does not directly examine the relationship between emotional polarization on social media and changes in user perspectives. To strengthen the validity of findings and provide empirical evidence, future research could incorporate qualitative methods such as surveys and interviews to gain deeper insights into how emotional discourse influences voter attitudes and decision-making.

## Figures and Tables

**Table 1:** Research on application of Twitter in elections (summary of research results).

**Category**	**Key Research Findings**
Political Parties/ Candidates	Opposition parties and candidates are more likely to use Twitter than members of the ruling party.
	Younger politicians are more willing to use Twitter compared to older generations.
	The extent of Twitter usage often correlates with the intensity of electoral competition.
	If a member of a political party has previously used Twitter successfully, other members of the party are more likely to adopt it.
	Political parties and candidates mainly use Twitter to share campaign information and links to their official websites.
	Calls for action, such as mobilizing voters, are relatively rare.
	Candidates from the ruling party tend to use Twitter in a broadcasting manner with limited interaction, while opposition candidates engage more actively with voters.
	Interactions between politicians mainly occur within the same party.
	Compared to television or newspaper, posting on Twitter increases candidates’ sense of connection and social presence.
	Candidates use Twitter to influence traditional media coverage of political issues.
	Candidates can use Twitter to raise small-scale political donations and increase campaign funding.
Public	Among users, only a small portion of the total population actively participates in election-related discussions on Twitter.
	Politically active users are mostly young, male, and students.
	Opposition party supporters are more likely than ruling party supporters to express opinions on Twitter and post more frequently.
	Twitter interactions show a political homophily pattern, where users engage mainly with those who share similar political beliefs, leading to intra-party communication.
	Supporters of different political parties tend to use distinct hashtags, creating politically segregated spaces for discussion.
	In terms of sentiment, most comments about candidates and political parties tend to be negative.
	The number of tweets generally increases over time, peaking near the election’s conclusion. Major political events related to candidates or campaigns also lead to sudden spikes in tweet volume.
	Tweet content mainly focuses on political leaders and their electoral actions rather than on key political issues or policy platforms.

**Table 2:** The SentimentWortschatz database corpus.

Word Category	Type	Positive Words	Negative Words
Adjective	Base Form	792	712
	Inflected Form	10,936	10,471
Adverb	Base Form	7	4
	Inflected Form	5	0
Noun	Base Form	548	688
	Inflected Form	736	1158
Verb	Base Form	297	423
	Inflected Form	3246	4580
Total	Base Form	1644	1827
	Inflected Form	14,923	16,209
Overall Total	—	16,567	18,036

**Table 3:** Sentiment value range and explanation of tweets.

Sentiment Score	Meaning and Examples
[−1, −0.5]	Indicates strong negative sentiment, such as criticism of candidates or policies, or offensive language.
[−0.5, 0]	Indicates mild negative sentiment, such as expressions of dissatisfaction or concern.
[0]	Represents neutral sentiment, typically used for factual descriptions, neutral opinions, or simple information.
[0, 0.5]	Indicates mild positive sentiment, such as expressions of support or a positive attitude.
[0.5, 1]	Indicates strong positive sentiment, such as enthusiastic support, excitement, or praise for candidates.

**Table 4:** Distribution of sentiment groups.

Group	N	Sentiment Score Ranges
Positive Group	400	(0, 1]
Neutral Group	400	[0]
Negative Group	400	[−1, 0)

**Table 5:** Descriptive Statistics of Sentiment Scores.

Indicator	Total Count	Mean	Standard Deviation	Minimum	25%	50% (Median)	75%	Maximum
Value	194,151	−0.3	0.41	−1	−0.6	−0.4	0	1

**Table 6:** Distribution of sentiment scores.

Sentiment Score	[−1, −0.5)	[−0.5, 0)	[0]	(0, 0.5]	(0.5, 1]
Count	94,253	41,688	19,932	31,361	6916
Proportion (%)	48.5	21.5	10.2	16.2	3.6

**Table 7:** Distribution of candidates’ sentiment scores.

Candidates	Mean	Positive (%)	Negative (%)
Baerbock	−0.372	2.6	46.13
Laschet	−0.325	4.24	45.58
Scholz	−0.225	8.38	32.35
Weidel	−0.275	4.24	32.68

**Table 8:** Negative lexicon table.

Candidate	High-Frequency Negative Words
Olaf Scholz	langweilig (boring), schweigt (silent), mechanisch (mechanical), zurückhaltend (reserved), passiv (passive), arrogant (arrogant), skandalös (scandal-ridden), reserviert (conservative)
Armin Laschet	inakzeptabel (unacceptable), posiert (pretentious), pietätlos (disrespectful, irreverent), grinsend (smirking), aalglatt (slick, cunning), unprofessionell (unprofessional), hinterhältig (insidious), sarkastisch (sarcastic), arrogant (arrogant), entscheidungsunfähig (indecisive), schwach (weak), unangemessen (inappropriate), zögerlich (hesitant), dominant (domineering), autoritär (authoritarian), manipulativ (manipulative), unbeliebt (unpopular), polarisierend (polarizing), frech (rude), kontrovers (controversial)
Annalena Baerbock	schlampig (messy, slovenly), debil (dumb), unehrlich (dishonest), hochstaplerin (hypocrite, fraud), arrogant (arrogant), naiv (naïve), inkompetent (incompetent), laut (loud), vorlaut (talkative), frech (rude), idealistisch (idealistic), schwach (weak), heuchlerisch (hypocritical), hysterisch (hysterical), unweiblich (not feminine enough), emotional (emotional), weibisch (effeminate), sensibel (overly sensitive, condescending towards women), kompetenzlos (incapable)
Alice Weidel	rechtsextrem (far-right extremist), nationalistisch (nationalist), spaltend (divisive), provokativ (provocative), populistisch (populist), polarisiert (polarized), radikal (radical), spaltend (creating division), empathielos (lacking empathy), provokant (provocative), unpopular (unpopular), hasserfüllt (full of hatred), unversöhnlich (uncompromising), kampfeslustig (belligerent)

**Table 9:** Kruskal–Wallis test results.

Kruskal–Wallis Test	
*p* value	<0.0001
*p* value summary	****
Do the medians vary signif. (*p* < 0.05)?	Yes
Number of groups	3
Kruskal–Wallis statistic	147.8

**** *p* < 0.0001.

**Table 10:** Dunn’s multiple comparison test results.

Dunn’s Multiple Comparisons Test	Mean Rank Diff.	Significant?	Summary	Adjusted *p* Value
Neutral vs. Positive	−220.3	Yes	****	<0.0001
Neutral vs. Negative	−283.7	Yes	****	<0.0001
Positive vs. Negative	−63.38	Yes	*	0.029

* *p* < 0.05; **** *p* < 0.0001.

**Table 11:** Dunn’s multiple comparison test details.

Test Details	Mean Rank 1	Mean Rank 2	Mean Rank Diff.	n1	n2	Z
Neutral vs. Positive	432.5	652.8	−220.3	400	400	8.993
Neutral vs. Negative	432.5	716.2	−283.7	400	400	11.58
Positive vs. Negative	652.8	716.2	−63.38	400	400	2.587

**Table 12:** Spearman correlation analysis results.

Spearman r	Negative (*n* = 400)	Positive (*n* = 400)
r	−0.2581	0.2523
95% confidence interval	−0.3499 to −0.1613	0.1553 to 0.3445
*p* (two-tailed)	<0.0001	<0.0001
*p* value summary	****	****
Exact or approximate *p* value?	Approximate	Approximate
Significant? (alpha = 0.05)	Yes	Yes

**** *p* < 0.0001.

**Table 13:** Linear regression analysis results.

Simple Linear Regression	Positive (*n* = 400)	Negative (*n* = 400)
Best-fit values		
Slope	80.96	−107.9
Y-intercept	12.99	−1.939
X-intercept	−0.1605	−0.01798
1/slope	0.01235	−0.009272
95% Confidence Intervals		
Slope	51.85 to 110.1	−139.7 to −75.97
Y-intercept	0.4000 to 25.59	−19.21 to 15.33
X-intercept	−0.4755 to −0.003772	−0.1408 to 0.1969
Goodness of Fit		
R2 (R squared)	0.06986	0.1
F	29.89	44.23
DFn, DFd	1, 398	1, 398
*p*	<0.0001	<0.0001
Deviation from zero?	Significant	Significant
Equation	Y = 80.96X + 12.99	Y = −107.9X − 1.939
Data		
Total values	400	400
Number of missing values	0	0

## Data Availability

All data generated or analyzed during this study are included in this article. The raw data are available from the corresponding author upon reasonable request.
